# Gradual hypertension induction in middle‐aged Cyp1a1‐Ren2 transgenic rats produces significant impairments in spatial learning

**DOI:** 10.14814/phy2.14010

**Published:** 2019-03-27

**Authors:** Mari N. Willeman, Monica K. Chawla, Marc A. Zempare, Lauren A Biwer, Lan T. Hoang, Ajay R. Uprety, Megan C. Fitzhugh, Matthew De Both, Paul D. Coleman, Theodore P. Trouard, Gene E. Alexander, Kenneth D. Mitchell, Carol A. Barnes, Taben M. Hale, Matthew Huentelman

**Affiliations:** ^1^ Evelyn F. McKnight Brain Institute University of Arizona Tucson Arizona; ^2^ Neurogenomics Division The Translational Genomics Research Institute (TGen) Phoenix Arizona; ^3^ Arizona Alzheimer's Consortium Phoenix Arizona; ^4^ Department of Basic Medical Sciences University of Arizona College of Medicine – Phoenix Phoenix Arizona; ^5^ Department of Psychology University of Arizona Tucson Arizona; ^6^ Center for Neurodegenerative Disease Research Biodesign Institute Arizona State University Tempe Arizona; ^7^ Department of Biomedical Engineering and Medical Imaging University of Arizona Tucson Arizona; ^8^ Neuroscience and Physiological Sciences Graduate Interdisciplinary Programs University of Arizona Tucson Arizona; ^9^ Department of Physiology Tulane University Health Sciences Center New Orleans Los Angeles

**Keywords:** Cognition, end organ damage, hypertension, renin angiotensin system

## Abstract

Hypertension is a major health concern in the developed world, and its prevalence increases with advancing age. The impact of hypertension on the function of the renal and cardiovascular systems is well studied; however, its influence on the brain regions important for cognition has garnered less attention. We utilized the Cyp1a1‐Ren2 xenobiotic‐inducible transgenic rat model to mimic both the age of onset and rate of induction of hypertension observed in humans. Male, 15‐month‐old transgenic rats were fed 0.15% indole‐3‐carbinol (I3C) chow to slowly induce renin‐dependent hypertension over a 6‐week period. Systolic blood pressure significantly increased, eventually reaching 200 mmHg by the end of the study period. In contrast, transgenic rats fed a control diet without I3C did not show significant changes in blood pressure (145 mmHg at the end of study). Hypertension was associated with cardiac, aortic, and renal hypertrophy as well as increased collagen deposition in the left ventricle and kidney of the I3C‐treated rats. Additionally, rats with hypertension showed reduced savings from prior spatial memory training when tested on the hippocampus‐dependent Morris swim task. Motor and sensory functions were found to be unaffected by induction of hypertension. Taken together, these data indicate a profound effect of hypertension not only on the cardiovascular‐renal axis but also on brain systems critically important for learning and memory. Future use of this model and approach may empower a more accurate investigation of the influence of aging on the systems responsible for cardiovascular, renal, and neurological health.

## Introduction

Hypertension is the leading risk factor for cardiovascular disease affecting 40% of middle‐aged adults 45–64 years and 70% of adults over the age of 65 in the United States (Gillespie and Hurvitz 2013). Wilkie and Eisdorfer (1971) first reported that the degree of hypertension in adults in their 60s was associated with significant intellectual decline over a 10‐year period. This general finding has been replicated and extended most recently in results from the Women's Health Initiative Memory Study, in which it was found that postmenopausal women with hypertension are at higher risk for cognitive decline (Haring et al. 2013). Other recent studies have also suggested that hypertension can impact cognitive function and brain structure even in the absence of stroke (Kilander et al. 1998; Harrington et al. 2000; Waldstein et al. 2008; Tsivgoulis et al. 2009) and it has been shown that gray matter volume as measured via magnetic resonance imaging can be reduced in the elderly with hypertension (Raz and Rodrigue 2006). The exact relationship between hypertension and cognitive decline, however, remains to be discovered.

A number of animal models have been developed to experimentally explore mechanisms through which hypertension has its impact on cognition. Among these include uninephrectomy‐induced hypertension (Jabaris et al. 2015a, 2015b), genetic models such as Dahl salt‐sensitive rats in which hypertension can be induced by a high sodium diet (Hirawa et al. 1999; Terry et al. 2001), chronic angiotensin II infusion to elevate blood pressure (Jing et al. 2012; Duchemin et al. 2013), and the spontaneously hypertensive rat (Wyss et al. 1992; Kantachuvesiri et al. 2001). There have, however, been a number of limitations to these models that make translation of the findings from these studies problematic. For example, experiments have been performed either in young hypertensive animal models, or older hypertensive rats in which blood pressure was elevated early in life. Given that hypertension tends to develop in middle age, an ideal model would use middle aged animals and a time course of hypertension induction that mimics known disease development more closely. A relatively new model of inducible renin‐dependent hypertension, the Cyp1a1‐Ren2 transgenic rat, allows the possibility of normal development and titrated induction of hypertension through the administration of a dietary‐based molecule (Meneses et al. 1996). This model is particularly powerful because it is based on the National Institute on Aging's colony of F344 rats which has produced the largest historical collection of data related to the study of normative aging in rats.

In this study, we more closely model human hypertension onset by inducing blood pressure increases in the middle aged Cyp1a1‐Ren2 rat. We investigate cardiac, renal, and vascular end organ changes elicited by the middle‐aged increase in blood pressure as well as the effects on spatial memory. We suggest that modeling hypertension in this approach has implications for the future investigation of the role of increased blood pressure on both peripheral and central organ systems.

## Methods

### Subjects

Fischer 344 rats made transgenic by the insertion of the mouse Ren2 renin gene fused to the cytochrome P450 1a1 promoter were used in these studies (Cyp1a1‐Ren2 (Mitchell et al. 2006)). In this model, the rats are fed aryl hydrocarbon indole‐3‐carbinol (I3C) that leads to the expression of Ren2 in the liver, increasing the circulating levels of renin and ultimately angiotensin II, resulting in elevated blood pressure. This dietary supplement can be added to rat chow in low levels, so that blood pressure is slowly increased over time. This makes it possible to regulate the rate and duration of blood pressure increases in rats in a way that mimics the development of hypertension in humans. Twenty‐five middle‐aged (15 months) male Cyp1a1‐Ren2 rats were used in this study, 13 that were given 0.15% I3C‐containing chow, and 12 that received the same chow without I3C (controls). I3C administration continued for 32 days. The animals were randomly assigned to groups. Chow was freely available throughout the study. Rats were single‐housed under a reverse light/dark cycle. This study was approved by the University of Arizona Institutional Animal Care and Use Committee and was conducted in accordance with the National Institutes of Health Guide for the Care and Use of Laboratory Animals. All rats used in this study were bred at Tulane University School of Medicine, New Orleans, LA.

### Blood pressure monitoring

Experimental blood pressure measurements were obtained from all animals on two occasions during the week prior to the addition of the I3C compound to the treatment group's chow. The device used for monitoring blood pressure was specifically designed for rodent use (CODA monitor and restrainer, noninvasive blood pressure monitoring system, Serial #SB0573, Kent Scientific). To obtain readings, animals were inserted into a snug plastic tube covered with a warming pad. Two cuffs (0.5 and 1.5 inches) were wrapped around the base of the animal's tail, and stable readings taken within 5–8 min of tube insertion. Animals were first adapted to blood pressure monitoring until they were comfortable with the procedure, which involved placing rats in the tube for ~15 min. For experimental measurements, three separate readings were taken on any given day to ensure equipment and animal stability.

### Behavioral testing procedures

Spatial learning and memory was assessed using the Morris swim task, which is a hippocampus‐dependent cognitive task. Experimenters were blinded to groups, and testing was randomized such that the hypertensive and control rats were not grouped when they were tested. The animals were tested throughout the same day. The detailed procedure for testing was as in Shen and Barnes (1996). Briefly, rats were given health checks and handled in the week before training. Rats were initially placed onto the hidden platform of the water tank for 30 sec, before being placed into the pool at pseudorandomly assigned start locations at the edge of the pool. A total of six spatial trials were given per day for 4 days (24 trials total). Following the spatial trials, six trials were given on each of 2 days (12 trials total) in which the submerged platform was above the water's surface (“cued trials”) to ensure that the animals did not have visual or motor defects that could influence performance. Since the start locations and swim velocity vary between animals, a corrected integrated path length (CIPL) is calculated to ensure comparability of the rats’ performance across release locations (Gallagher et al. 1993). A two‐way repeated measures ANOVA was used to assess differences between treatment groups over training trials before the hypertension treatment began, with alpha levels set at 0.05. The retest procedure was identical to the initial Morris swim task test, including 24 spatial trials over 4 days (with the platform in the same location) and 12 cued trials over 2 days (in order to determine if the blood pressure modification impacted motor or visual capacity). An additional dependent measure for assessing the spatial learning variable involves how much “savings” occurred in individual rats between the initial training (before hypertension was induced) and the “retest” after induction of hypertension. This was calculated as the difference in performance for each rat over each trial (“Pre–Post”, so that it would be a positive value) normalized by dividing by “Pre + Post”. The statistics used for this comparison was a mixed effect model, with treatment as the random effects variable and test day and group as the fixed variables.

## Experimental Procedure

Rats were shipped from Tulane to Tucson at ages ranging from 6 to 10 months. They were quarantined for 6–8 weeks in Tucson. After quarantine was complete, the rats were handled, weighed, and grossly examined beginning 2 weeks before the start of the experiment. The initial behavioral assessment, using the Morris swim task, was given over a 1 week period. Six days after this first behavior test, diastolic and systolic blood pressure measurements were begun 2 days prior to beginning the I3C or control diet. Treatment was given for 28 days. The behavior retest began at day 30 for the final phase of the experiment (i.e. post‐treatment behavior “retest” commenced 32 days after treatment onset). Following the final behavioral test, the animals were euthanized and organs were collected for analysis.

In additional to the transgenic rats tested, we fed the I3C and control diet to eight wildtype F344 rats (four rats were given I3C diet, four rats were given the control diet). The procedure was identical to that described above for the transgenic F344 rats. This allowed us to determine whether the diet impacted behavioral performance without the transgene present.

### Heart and kidney weight measurements

Heart and left kidney were rapidly dissected for analysis of organ weight. The ureter, renal vein, and renal artery were removed as closely as possible to the renal capsule. All connective (fascia) and adipose tissue (perirenal fat) contacting the renal capsule was carefully removed. The kidney was rinsed with 0.9% NaCl in water and dried between paper towels. After 2 min of drying the kidney was weighed. The vena cava, aorta (including aortic arch), and pulmonary vessels were removed from the heart as closely as possible to the base of the ventricles. The heart was immediately immersed in 0.9% NaCl in water to enable the clearance of as much blood as possible from the ventricles. The heart was then blotted dry between paper towels and allowed to dry for 2 min before being weighed. Body weight of each animal was collected at sacrifice and utilized in the analysis of heart and kidney weights. A Wilcoxon rank‐sum (WRS) test was utilized to identify statistically significant differences (*P* < 0.05).

### Trichrome staining and analysis

Whole mount heart tissue was immersion‐fixed in 4% paraformaldehyde with PBS for 16 h at 4°C, rinsed briefly in PBS three times to remove residual paraformaldehyde, and stored in 30% sucrose with PBS at 4°C for 48 h before being embedded in Tissue‐Tek O.C.T Compound (Sakura Finetek) in a transverse orientation for cryosectioning. The frozen heart tissue was sliced in 6 *μ*m sections. A minimum of four sections were collected per animal. Microwave Masson's Trichrome Stain Kit Procedure (American MasterTech) was used to stain the heart sections of each animal for collagen. Pictures of each stained section were taken at 10× using a Zeiss Axio epifluorescent microscope, an Axiocam HRc, and Axiovision software. The pictures were taken while blinded to the group, such that there was no information regarding the hypertensive status of the animal. Sections were thresholded for red (cytoplasm control) or blue (fibrotic collagen deposits) using ImageJ's (http://rsbweb.nih.gov/ij/) Threshold Color plug‐in (http://www.dentistry.bham.ac.uk/landinig/software/software.html) and analyzed with the histogram tool at Hue = 0 (black). Red was defined at the hues 205–255, and blue was defined as the hues 150–200. The data were analyzed for outliers using the Grubb's test at a significance level of 0.05, and one outlier was found and removed from analysis. A Wilcoxon rank‐sum (WRS) test was utilized to identify statistically significant differences (*P* < 0.05).

### Vascular end organ damage measurements

Paraformaldehyde (4%) fixed transverse sections of thoracic aorta (segment obtained between the 2nd and 3rd intercostal) were dehydrated with graded ethanol solutions, embedded in paraffin and sectioned at 5 *μ*m. Sections were rehydrated and stained with orcein to identify elastic fibers. Morphological assessments were made in 40× magnification images using Image J software for determination of the cross sectional area for the medial layer of the aorta, lumen diameter, medial wall thickness, and media‐to‐lumen ratio. Cellular proliferation was evaluated in separate cells based on proliferating cell nuclear antigen (PCNA) expression, as described previously (Biwer et al. 2016). Briefly, following rehydration and antigen retrieval in a 0.01 mol/L Tris‐HCl pH 8.6 buffer in a microwave pressure cooker, sections were blocked (5% normal goat serum), and incubated overnight at 4°C with monoclonal mouse anti‐PCNA (1:500, Dako), followed with anti‐mouse secondary (1:200, Dako) and Streptavidin‐HRP (1:400, Dako). Positive staining was visualized as a brown precipitate after addition of diaminobenzidine (DAB). Cells positive for PCNA were counted and normalized to the cross sectional area by a blinded observer. A Wilcoxon rank‐sum (WRS) test was utilized to identify statistically significant differences (*P* < 0.05).

## Results

### Blood pressure measurements

**Figure 1:**
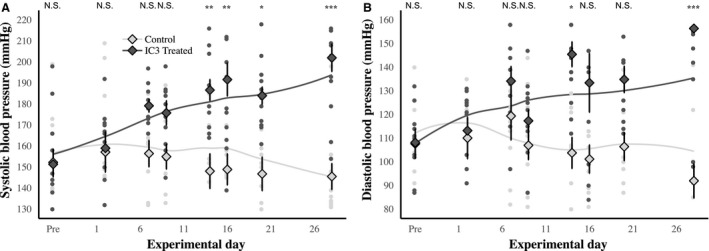
I3C treatment significantly increases blood pressure. I3C treatment significantly increases both (A) systolic and (B) diastolic blood pressure. Indirect blood pressures were measured using the tail‐cuff method approximately once each week. I3C‐treated animals (*n* = 13) demonstrated a time dependent increase in blood pressure throughout the experimental period. Small filled circles are the measured values for individual animals in each group (dark gray: I3C treated, light gray: Control, *n* = 12). Diamonds are the group means and the error bars are the standard deviation of the means. Linear model fit lines are included. These results demonstrate that the I3C treatment was sufficient to increase blood pressure in the Cyp1a1‐Ren2 animals. Two‐way repeated measures ANOVA was performed and the post hoc *P*‐value for Experimental Day is indicated at the top of the plot (*P < 0.05, **P < 0.01, ***P < 0.0001, N.S. = not significant, *P* > 0.05).

Animals (*n* = 25 total) were raised on normal control chow until they were 15 months old. Prior to hypertension induction with I3C, baseline indirect blood pressure measurements were obtained, and no statistically significant differences between the treatment and control groups were noted (151/108 vs. 152/107) (Fig. 1). Indirect systolic and diastolic blood pressures increased significantly, however, (with significant differences noted for systolic and diastolic after 14 and 28 days respectively) over the course of the 28‐day induction period in I3C exposed rats. At day 28, systolic blood pressure in the I3C treated animals (*n* = 13) had reached 195 mmHg (Con: 145 mmHg) on average and diastolic reached an average of 150 mmHg (Con: 92 mmHg) (Fig. 3). We did not record heart rate; however, others have measured heart rate in this same animal model during I3C induction of hypertension and noted no significant differences (Heijnen et al. 2014).

### Behavioral characterization

**Figure 2:**
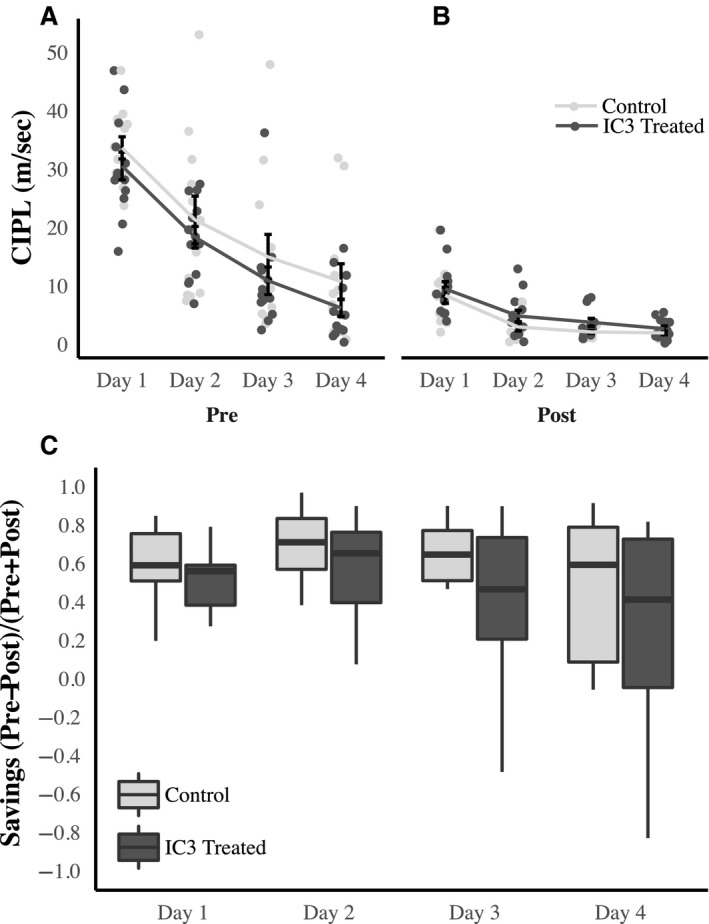
Hypertension reduced spatial memory performance on the Morris water maze. Spatial memory as assessed by Morris water maze performance was measured before (PRE) and after (POST) long‐term treatment with I3C (*n* = 13) or Control (*n* = 12) chow (dark gray: I3C treated, light gray: Control). Animals were randomly assigned to treatment groups; however, we noted a trend in the control chow‐assigned animals to perform worse during the PRE phase. Thus we calculated a Cognitive Savings score for each individual animal (the difference in performance between the PRE and POST tasks). We used a mixed effect model with animal ID as a random effects variable and both test day and group (treatment vs. control group) as fixed effects variables. Treatment affected the Cognitive Savings score (*χ* ^2^(1) = 4.11, *P* = 0.04), with treated animals experiencing less savings 0.17 ± 0.079 (SEM) than controls. These results demonstrate that the I3C treatment impaired spatial reference memory performance in the Cyp1a1‐Ren2 animals.

Learning and memory performance was tested using the Morris swim task, both before (“PRE”) and after (“POST”) treatment with I3C‐chow. The performance of the control and hypertensive rats before treatment is shown in Figure 2A, which illustrates that there were no statistically significant differences between groups at the beginning of the experiment (*F*_23,69_ = 0.09, *P* = >0.05). After treatment, there were still no significant difference between the treated and control rats (Fig. 2B, *F*_23,69_ = 0.20, *P* = >0.05). Because there was a trend in the pretreatment I3C group to perform better on the spatial memory task than the randomly assigned controls, we calculated a “cognitive savings” score for each individual animal for the spatial condition. This score allows a determination to be made, within a given rat, of how much the first 24 training trials impacted performance on the second set of 24 trials. This number was obtained by first calculating the CIPL value difference between the pretreatment and post treatment watermaze data for each individual rat on each trial. The data were then normalized for each individual rat across all 4 days, by the following formula: (pre‐post/pre + post). These values were then averaged for each animal in each group (control or hypertension) to obtain a savings score for each day and each treatment group. The CIPL score decreased for both groups during the “post” testing sessions. The significant effect of group in the mixed model shows that there were larger differences in CIPL values between the initial learning and relearning trials in the control group, compared to the treated animals. Our hypothesis was that induction of hypertension would impair the relearning process, thus resulting in lower “savings” scores in the treated animals. To test whether treatment affected cognitive savings, a mixed effect model was employed with animal ID as a random effects variable and both test day and group (treatment vs. control group) as fixed effects variables. Treatment significantly affected savings (*χ* ^2^(1) = 4.11, *P* = 0.04), with the hypertension group not benefiting as much from initial training as did the controls (Fig. 2C “Cognitive Savings”), with treated animals experiencing lower savings (0.17 ± 0.079) than controls. The 0.17 value is the average difference between controls and treated animals across “days,” indicating that savings is greater in control animals compared to treated animals. The standard error value (0.079) is the standard deviation of differences between controls and treated across “days.” The reduced savings in the I3C‐treated rats could be explained by faster forgetting of the first set training trials, poorer relearning of the second set of training trials, or a combination of these factors.

**Figure 3:**
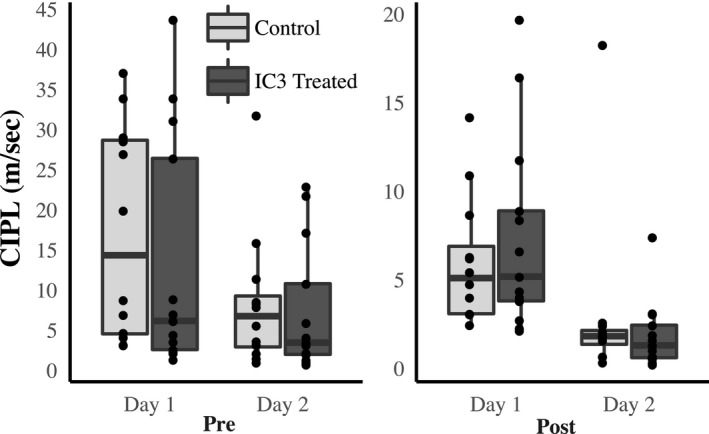
Hypertension has no effect on visual cued Morris swim task performance. A visual cued version of the Morris swim task was performed during the PRE and POST phases of the experiment (dark gray: I3C treated, *n* = 13; light gray: Control, *n* = 12). There were no statistically significant differences detected due to I3C treatment. These results demonstrate that hypertension did not significantly impair vision or alter swimming speed in the Cyp1a1‐Ren2 animals.

On the other hand, for the cued version of the swim task, there were no differences, nor trends, between control or treatment groups in either the PRE or POST phases (*F*_23,23_ = 0.56, *P* = >0.05; *F*_23,23_ = 2.7, *P* = >0.05, Fig. 3. This indicates that sensory or motor components that contribute to task performance cannot account for the cognitive changes observed in spatial behavior.

In addition to administering the I3C diet to the transgeneic F344 rats, we tested the potential impact of the I3C diet on wildtype F344 rats. We gave four wild type rats the I3C diet and four wild type rats the global 18% protein diet for 4 weeks. Systolic and diastolic BP and body weights were measured during the 28 days of treatment, as well as during the following week of final behavior retest. A two‐way ANOVA showed no significant difference between the control and I3C rats in body weight – *F* (1,6), *P* = 0.92, in spatial behavior – *F* (1,31) = 0.26, *P* = 0.61, or in systolic BP – *F* (1,6) = 2.433, *P* = 0.17, or diastolic BP – *F* (1,6) = 5.86, *P* = 0.052.

### Pathological characterization

**Figure 4:**
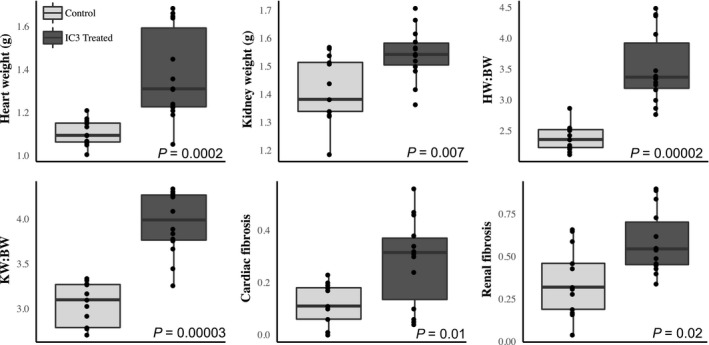
I3C treatment significantly increases cardiac and renal measurements of end organ damage. End organ damage was measured in each animal at sacrifice. I3C (*n* = 13) treated animals are shown here as dark gray, and control (*n* = 12) animals as light gray. These results demonstrate that the I3C induction of hypertension was sufficient to increase common measures of cardiac and renal end organ damage in the Cyp1a1‐Ren2 animals. Wilcoxon ranked sum test *P*‐values are indicated for each comparison.

**Figure 5:**
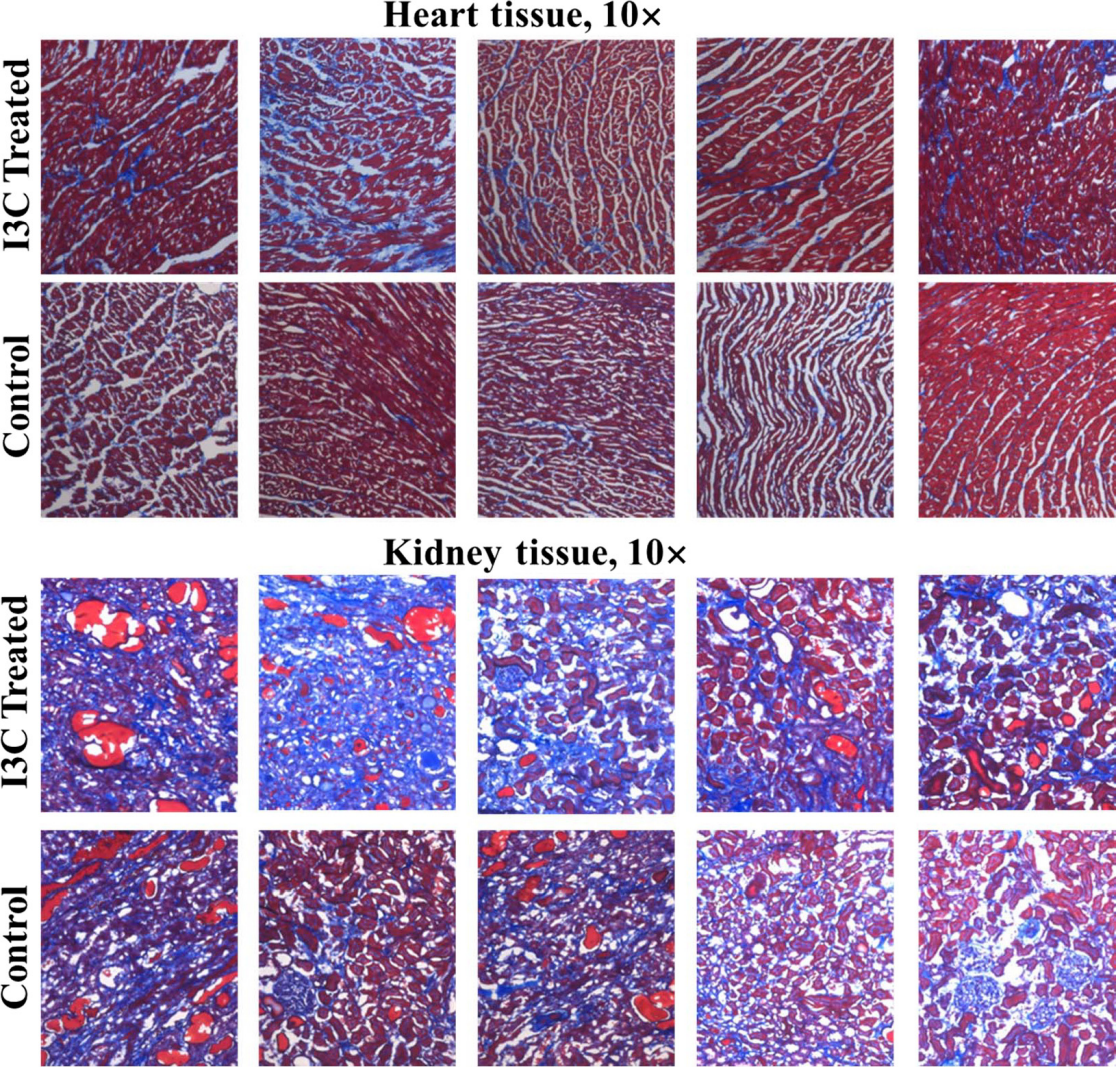
Hypertension results in significantly elevated cardiac and renal fibrosis. Representative images from heart and kidney tissue using Masson's trichrome stain to indicate collagen fibrosis. Images that scored nearest the group means are illustrated here.

**Figure 6:**
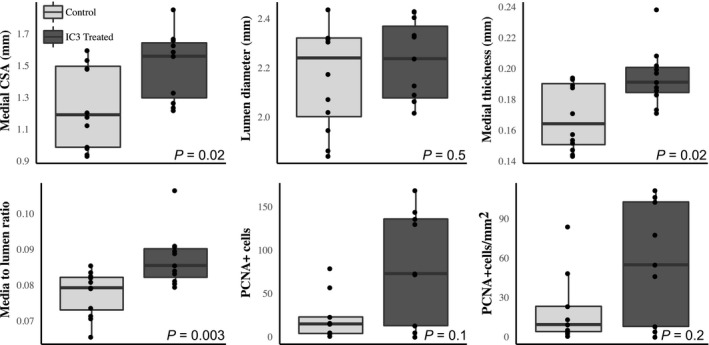
Hypertension induces end organ damage in the aorta. I3C (*n* = 13) treatment induced aortic hypertrophy as evidenced by increases in medial cross‐sectional area (CSA), medial thickness, and the media to lumen ratio. Increased cellular proliferation was evaluated based on the number of cells positively labeled with anti‐proliferating cell nuclear antigen (PCNA). Wilcoxon ranked sum test *P*‐values are indicated for each comparison.

At sacrifice cardiac and renal measures of end organ damage were assessed. Heart weight (*W* = 169, *P* = 0.0002), kidney weight (*W* = 147, *P* = 0.007), heart weight to body weight ratio (HW:BW, *W* = 180.5, *P* = 1.6E‐05), kidney weight to body weight ratio (KW:BW, *W* = 178, *P* = 2.7E‐05), cardiac fibrosis (*W* = 144.5, *P* = 0.01), and renal fibrosis (*W* = 141.5, *P* = 0.02) were all significantly different versus the control chow animals (Wilcoxon ranked sum test *P*‐values, Fig. 4). Slightly larger effect sizes for all end organ damage measurements were noted for the heart. Figure 5 illustrates representative examples of Masson's trichrome staining of cardiac and renal tissue demonstrating enhanced collagen deposition in I3C treated rats. Figure 6 demonstrates the end organ damage measurements performed on the thoracic aorta. The I3C‐treated rats had marked histological and proliferative changes in their aortas compared to control rats. Medial cross sectional area, medial thickness, and media to lumen ratio were significantly increased (*W* = 103, *P* = 0.02, *W* = 105, *P* = 0.02, and *W* = 115, *P* = 0.003 respectively) while lumen diameter did not change (*W* = 77, *P* = 0.5). Associated with the increased medial thickness was an increase in the number of proliferating cells in I3C aortas (total cell counts: *W* = 59.5, *P* = 0.1 versus control, cells per mm^2^: *W* = 56, *P* = 0.2).

**Figure 7:**
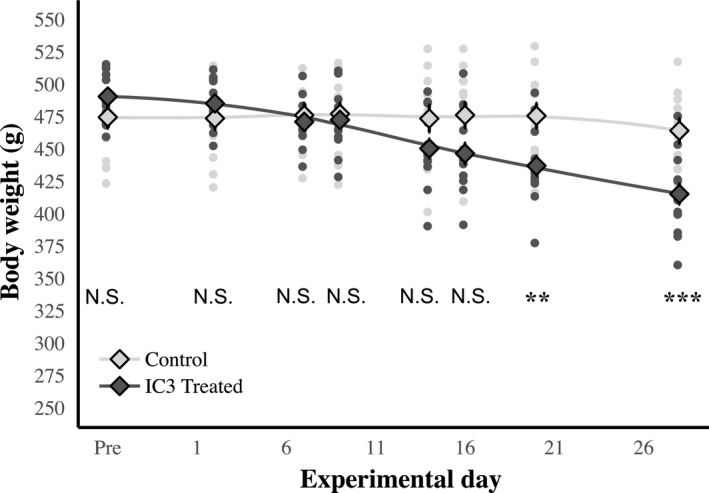
I3C treatment is associated with significant body weight loss after 2 weeks. The I3C (*n* = 13) treated animals have significant drops in body weight by the end of the experimental period. Post hoc analysis suggests that weight tends to diverge by day 14 (**P < 0.01, ***P < 0.001, N.S. = not significant).

Body weight measurements were also obtained before and throughout the treatment protocol (Fig. 7). There was a significant main effect of treatment (*F*_15,140_ = 24.85 *P* < 0.0001). Post hoc analysis indicates that the weights tend to diverge by day 14 of I3C ingestion, which persisted until the end of the study.

**Figure 8:**
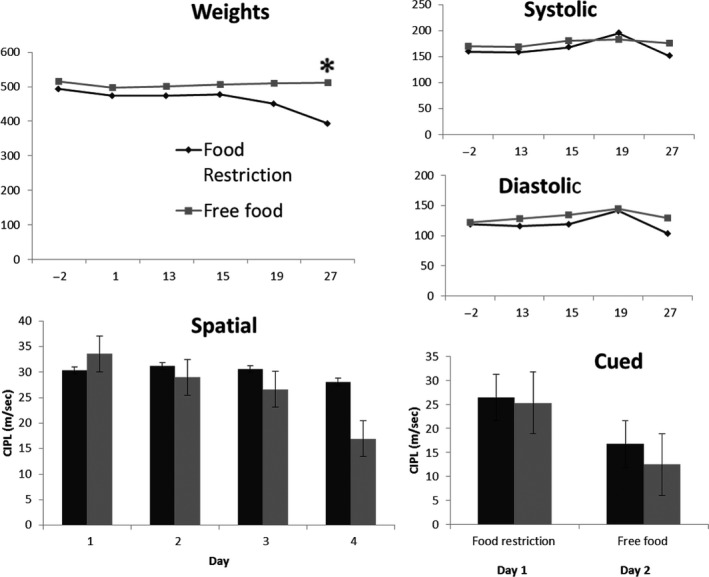
Weight loss results in no significant changes in spatial or cued behavior, and treatment did not affect blood pressure. The food restricted group (*n* = 3) did show significant weight loss by two‐way repeated measures ANOVA, (*F*_[28,140]_ = 24.90, *P* < 0.001). Post hoc analysis revealed a significance difference at day 27 (*), *P* < 0.001). No significant differences in systolic or diastolic blood pressures were observed between groups, nor were there differences in the spatial or cued versions of the Morris swim task (*P* > 0.05).

To address any concerns that weight loss may have had a negative impact on our cognitive measures, a food restriction study was performed in wild type F344 rats (*n* = 3 food restricted; *n* = 3 ad libitum). The body weights in the restricted group were decreased to the same amount as the I3C‐treated animals beginning in the second week of the experiment, and continued for 27 days. There was a main effect of treatment on weight (*F*_28,240_ = 24.90, *P* < 0.001), with post hoc analysis indicating a significant difference by day 27 (*P* < 0.001). There was no main effect of treatment on blood pressure or on cognition between groups (*P* > 0.05; Fig. 8).

## Discussion

The experiments in this study examined the cardiovascular, renal, and neurological effects of hypertension induction in middle‐aged rats. Our approach included a slow induction of hypertension at an age in the rat that is equivalent to the human manifestation of primary hypertension. Middle‐aged induction with a slow rise in blood pressure was our attempt to more closely model the human disease. Inducing hypertension in this fashion resulted in significant changes in systolic and diastolic blood pressures (Fig. 1) as well as cardiovascular and renal end organ damage (Figs. 4, 5, 6). We recognize that the control diet fed animals also demonstrated a slightly elevated blood pressure as well, and this change may be age‐related as others have found in studies utilizing rats (Buñag and Teräväinen 1991). Additionally, we observed a small but statistically significant reduction in the ability of rats to benefit from spatial learning pretraining, when spatial memory was tested following hypertension induction (Fig. 3). Hypertension induction, however, had no effect on visual discrimination or swim speed (Fig. 2). In summary, modeling hypertension in this fashion produced much of the predicted peripheral, as well as the less well understood cognitive effects of hypertension.

We noted a potential confound in our model related to weight loss, potentially due to reduced food intake (Fig. 7), although this was not directly investigated. Since weight loss could indicate suboptimal nutrition and that in turn could influence cognitive status we performed a control experiment where food deprivation was used to cause a longitudinal drop in body weight. In the food deprived animals we did not note an influence on cognitive performance in the Morris water maze (Fig. 8) suggesting that the significant changes we noted in the animals with hypertension were not related to their gradual weight loss and are most likely a consequence of the increased activation of the renin angiotensin system.

We propose that processes associated with aging should be considered when hypertension is modeled in the rodent. In the human condition, prehypertension and hypertension occur in midlife. Hypertension in midlife has been associated with a higher risk of dementia or poor cognitive function later in life (Launer 1995; Yano et al. 2014; Gottesman et al. 2017). The Cyp1a1‐Ren2 rat model has not previously been used to study the effects of hypertension on cognition. Many of the approaches in the field induce hypertension in animals that are younger than the equivalent human patient and they also tend to utilize very rapid induction approaches that result in a rise in blood pressure that is estimated to be much steeper than is typically the case in humans. Of note, the Cyp1a1‐Ren2 rat is well suited for the study of age‐related changes and hypertension since the model is based on the Fisher‐344 rat, a rodent model developed by the National Institute on Aging at the NIH. Because of this fact, there are many studies that have examined aging as well as cognition in this background strain that can be used as appropriate starting points for future study. The noninvasiveness of the model is also a positive aspect that helps facilitate its use in the study of aging. Without the need for surgical intervention there are generally fewer complications associated with the model. Additionally the use of a xenobiotic induction approach lends itself well to aligning with an ideal antihypertensive therapy. That is, animals can simply be switched to normal chow, which in turn shuts off the expression of the renin transgene, to evaluate the impact of removing the initial hypertensive stimulus.

The underlying mechanism responsible for the impairment in the ability of rats to benefit from spatial learning pretraining in this study remains to be determined. In addition to hypertension and upregulation of the renin angiotensin system, systemic inflammation secondary to target organ damage have each been associated with impaired cognitive function (Guo et al. 2017; Yamanaka et al. 2017; Ahmed et al. 2018; Dhanda et al. 2018). Central inflammation is likely not responsible for the effects seen in the present manuscript as others have shown that greater than 4 months of I3C treatment in Cyp1a1‐Ren2 rats was required to induce an increase in microglia (Pannozzo et al. 2013). Given the modest effects that we observed after only 32 days of I3C treatment, it would be interesting to see the effects of long‐term induction on cognition using this model. Long‐term induction of hypertension in aged Cyp1a1‐Ren2 rats with a high‐fat diet have been shown to have microstructural alterations but no white matter lesions (Holland et al. 2015). These modest changes, as well as the modest changes seen in our work, are an indication that this model could be beneficial in observing the mechanisms at play in the human condition.

Limitations in our study include, first, the weight loss associated with the xenobiotic diet. We controlled for the weight loss but could not control for other unrecognized effects that the diet may have had on the animal. We did, however, characterize swimming speeds and vision in the Morris swim task, and both of those measurements were equivalent between treated and untreated animals. We also treated nontransgenic F344 rats with I3C and control diet to examine the potential effect of this xenobiotic compound on body weight, blood pressure, and spatial behavior. There was no impact of I3C on any of these variables, suggesting that the xenobiotic diet only had an effect on the transgenic animals. Secondly, blood pressure measurements were conducted using indirect approaches. Our use of indirect blood pressure measurements instead of surgical intervention on the middle‐aged animals likely resulted in a larger standard deviation for the blood pressure measurements. Regardless of this, the induced animals demonstrated a significant rise in both systolic and diastolic pressures compared to the untreated rats. Lastly, only spatial reference memory was assessed in the animals. Future work should explore a more detailed battery of behavior tasks to examine what cognitive domains are most strongly influenced by hypertension. A battery of assessments is used to determine cognitive function when studying the effects of hypertension on cognition in humans (Iadecola et al. 2016), and it would be beneficial to include a battery that closely mimics the assessments in animal work as well.

In summary, we utilized an inducible model of hypertension and attempted to closely mimic both the age‐at‐onset and slow blood pressure rise associated with human primary hypertension. Induction of hypertension resulted in predicted changes to cardiac, renal, and vascular end organs as well as to spatial reference memory performance. These findings suggest that the Cyp1a1‐Ren2 rat model is amenable to the study of hypertension in middle age and that the phenotype mimics both the peripheral and central alterations associated with elevated blood pressure that is noted in the human.

## Conflict of Interest

None declared.
